# Extracorporeal Membrane Oxygenation-Assisted Thoracic Surgery: A Series of 10 Cases

**DOI:** 10.70352/scrj.cr.24-0004

**Published:** 2025-03-15

**Authors:** Yuzu Harata, Kazuhiro Imai, Shinogu Takashima, Shoji Kuriyama, Hidenobu Iwai, Haruka Suzuki, Ryo Demura, Sumire Shibano, Yoshihiro Minamiya

**Affiliations:** Department of Thoracic Surgery, Akita University Graduate School of Medicine, Akita, Akita, Japan

**Keywords:** extracorporeal membrane oxygenation, thoracic surgery, anticoagulation, airway obstruction

## Abstract

**INTRODUCTION:**

Extracorporeal membrane oxygenation (ECMO) is a type of extracorporeal circulation used to divert blood from and deliver blood to peripheral blood vessels. Recently, the use of ECMO has been reported in various non-transplant surgeries. Particularly in tracheal surgeries, ECMO provides an unobstructed surgical field and enables safe induction of general anesthesia in difficult intubation cases. Here, we report on 10 cases of thoracic surgery in which ECMO was employed at our institution.

**CASE PRESENTATION:**

These 10 cases comprise 4 tracheal cancer surgeries, 2 lung cancer surgeries, and 1 case each of surgery for thyroid cancer, mediastinal cancer, tracheomalacia, and tracheobronchial injury. Veno-venous (VV)-ECMO is most often selected, but veno-arterial (VA)-ECMO is chosen when recirculation with VV-ECMO is unacceptable, when pulmonary artery bleeding needs to be controlled, or when cardiac support is necessary. Among the 10 presented cases, VV-ECMO was used in 8, while VA-ECMO was employed in 2. Three of these cases involved ECMO bailout due to dyspnea caused by airway stenosis. Six of the patients did not receive heparin maintenance. Of those, 1 was maintained on nafamostat mesilate, 2 were maintained on nafamostat mesilate after receiving a single dose of heparin, and 3 received only a single dose of heparin. In none of those cases did ECMO fail to maintain flow due to thrombus formation. A postoperative hemothorax occurred as one of the ECMO-related complications in Case 4. There were no perioperative cardiopulmonary complications, in-hospital deaths, or deaths within 30 days after surgery. One patient died from metastatic recurrence of non-small cell lung cancer 5 months after surgery, another from progression of disease in mediastinal anaplastic cancer 4 months after surgery, and the 3rd from upper gastrointestinal bleeding 2 years after surgery. The other 7 patients remain alive.

**CONCLUSIONS:**

ECMO is useful in tracheal surgery and in cases where intubation is difficult or dangerous, because it facilitates safe and accurate surgery. We also believe that individualized anticoagulant strategies can be safely implemented.

## Abbreviations

ACT
: activated clotting time
APC
: argon plasma coagulation
ASA-PS
: American Society of Anesthesiologists-Physical Status
CT
: computed tomography
ECLS
: extracorporeal life support
ECMO
: extracorporeal membrane oxygenation
ELSO
: Extracorporeal Life Support Organization
LVEF
: left ventricular ejection fraction
PA
: pulmonary artery
SIRS
: systemic inflammatory response syndrome
VA
: veno-arterial
VV
: veno-venous
VVA
: veno-veno-arterial

## INTRODUCTION

Extracorporeal membrane oxygenation (ECMO) is a type of extracorporeal circulation that diverts blood from and delivers blood to peripheral blood vessels. Although ECMO is often used in cardiac surgery, lung transplant surgery,^1)^ and trauma surgery,^2)^ there have been few reports of its use in non-transplant thoracic surgery. Some have expressed concern that ECMO may increase the risk of hemorrhagic complications and the possibility of tumor cell dissemination.^3)^ Recently, however, the use of ECMO has been reported in thoracic surgeries where oxygenation cannot be maintained with normal isolated lung ventilation.^3,4)^ In tracheal surgeries, ECMO provides an unobstructed surgical field, enabling accurate operation^4–7)^ and safe induction of anesthesia in cases where intubation is difficult or breathing is spontaneous. The risk of catastrophic bleeding cannot be ignored in any thoracic surgery, but preoperative risk identification and selection of an optimal anticoagulation strategy can minimize bleeding events.^8–11)^ This case series study aims to retrospectively examine the use of ECMO in non-transplant thoracic and respiratory surgeries for which no clear consensus has been reached, and to identify problems and points to consider when performing the procedure in practice.

## CASE PRESENTATION

### Patients

This retrospective case series involved 10 consecutive patients who underwent thoracic surgery with ECMO at Akita University Hospital between 2020 and 2023. These included cases in which intraoperative airway clearance was difficult, such as tracheal surgery; cases in which ventilation failure was expected due to pneumonia or postoperative lung volume loss in the contralateral lung; cases in which there was a risk of low cardiac output due to compression by a large mass; and cases in which pulmonary artery (PA) hemorrhage had to be controlled. Patients who were expected to experience ECMO weaning failure postoperatively,^3)^ such as those with severe postoperative lung dysfunction, irreversible pneumonia, heart failure, or other multiple organ failure, were excluded.^12–14)^

### ECMO protocol

**Fig. 1:**
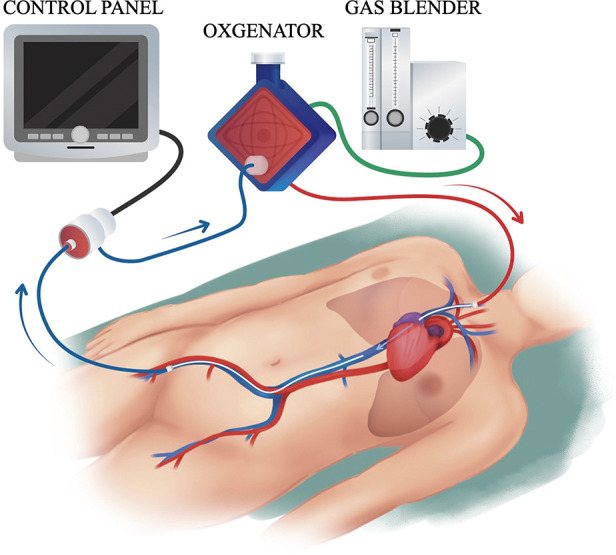
Typical insertion sites are the right femoral vein for the drainage cannula and the right jugular vein for the return cannula. ECMO, extracorporeal membrane oxygenation

**Fig. 2:**
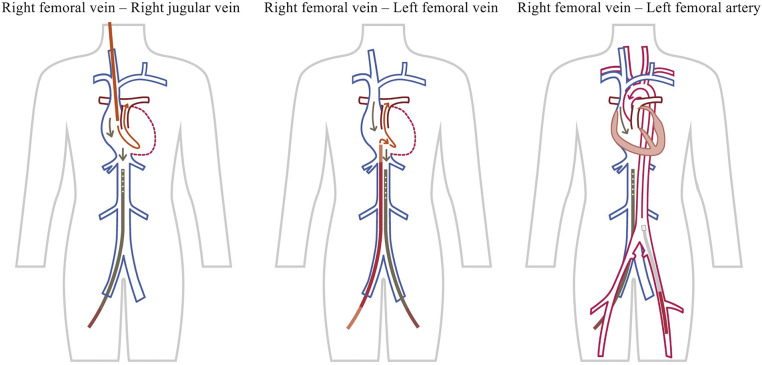
If there is a possibility of constricting or blocking the right jugular vein or superior vena cava, the right subclavian vein, the left femoral vein, or the left femoral artery (VA-ECMO) are also options. VA-ECMO, veno-arterial extracorporeal membrane oxygenation

Venous-venous (VV)-ECMO was selected in most cases. The indication for veno-arterial (VA)- or VV-ECMO depended on the organ being supported: either cardiac and/or respiratory support. VA-ECMO was used in cases of hemodynamic instability (Case 8), heart failure, or a high risk of PA bleeding, such as in a hilar lung cancer case where one of the main PAs needed to be clamped (Case 5). ECMO was introduced using a protocol that followed Extracorporeal Life Support (ECLS) Organization (ELSO) guidelines.^14)^ The insertion site was normally the right femoral vein for the drainage cannula and the right jugular vein for the return cannula (**Fig. 1**). However, if there was a possibility of constricting or blocking the right internal jugular vein or superior vena cava, the right subclavian vein, the left femoral vein, or the left femoral artery were also options (**Fig. 2**). In rare cases of VV-ECMO, recirculation—where pumped, oxygenated blood flows directly to the drainage cannula without circulating through the body—was a major concern. Poor oxygenation with recirculation was addressed by adjusting the position of the cannula tip while monitoring the SpO2 of the drainage blood.^15,16)^ If that did not work, another return cannula was inserted into the artery (e.g., femoral artery; veno-veno-arterial [VVA]-ECMO). Otherwise, in the case of poor drainage due to constriction of the inferior vena cava, the insertion sites of the drainage and return cannulas were exchanged. We have no experience with single-site dual-lumen cannulation at our institution.^17)^ As an anticoagulant strategy, heparinization or maintenance with nafamostat mesilate was used in patients at high thrombotic risk, while only a single dose of heparin was used in patients at high risk of bleeding. With heparin or nafamostat mesilate maintenance, activated clotting time (ACT) was set to around 200 s. After the cannula was removed, protamine was administered to the heparinized patients.

### The specific surgical procedure

In preparation for ECMO, all patients received whole-body contrast-enhanced computed tomography (CT), a blood coagulation test, cardiac ultrasonography, and blood gas analysis. Before surgery, patients were positioned supine for the placement of the cannulas for the ECMO circuit. If intubation was possible, ECMO was introduced after general anesthesia. Cannulas were inserted using a percutaneous technique, preferably guided by ultrasonography, performed by board-certified cardiac surgeons with expertise in both percutaneous and open procedures. The insertion site of the central venous catheter and monitoring position were adjusted accordingly. When changing the patient’s position, careful attention was paid to ensure that the position of the cannula did not change. Pulmonary ventilation was then temporarily stopped, after which the amount of recirculation was estimated based on the oxygen level in the drainage blood, and the position of the cannula tip was adjusted. In addition, cerebral blood flow was checked using cerebral oximetry (rSO_2_), which noninvasively measures brain tissue oxygen saturation with near-infrared spectroscopy (INVOS 5100 C system; Medtronic, Minneapolis, MN, USA). We assessed cerebral blood flow and checked systemic oxygenation using an A-line inserted in the right upper extremity and SpO_2_ monitors attached to the upper and lower extremities. Pulmonary ventilation was resumed after confirming that it was manageable. Thereafter, respiratory arrest was only initiated if deemed necessary. In cases where tracheal intubation was difficult due to tracheal obstruction, cannulation was performed under local anesthesia, and ECMO circulation was initiated while the patient breathed spontaneously. This was followed by tracheal intubation or the use of a laryngeal mask. After the surgery, the cannula was removed with the patient in a supine position, and bleeding at the puncture site was stopped with pressure hemostasis or a suture stitch with 3-0 VICRYL.

### Cases

**Fig. 3:**
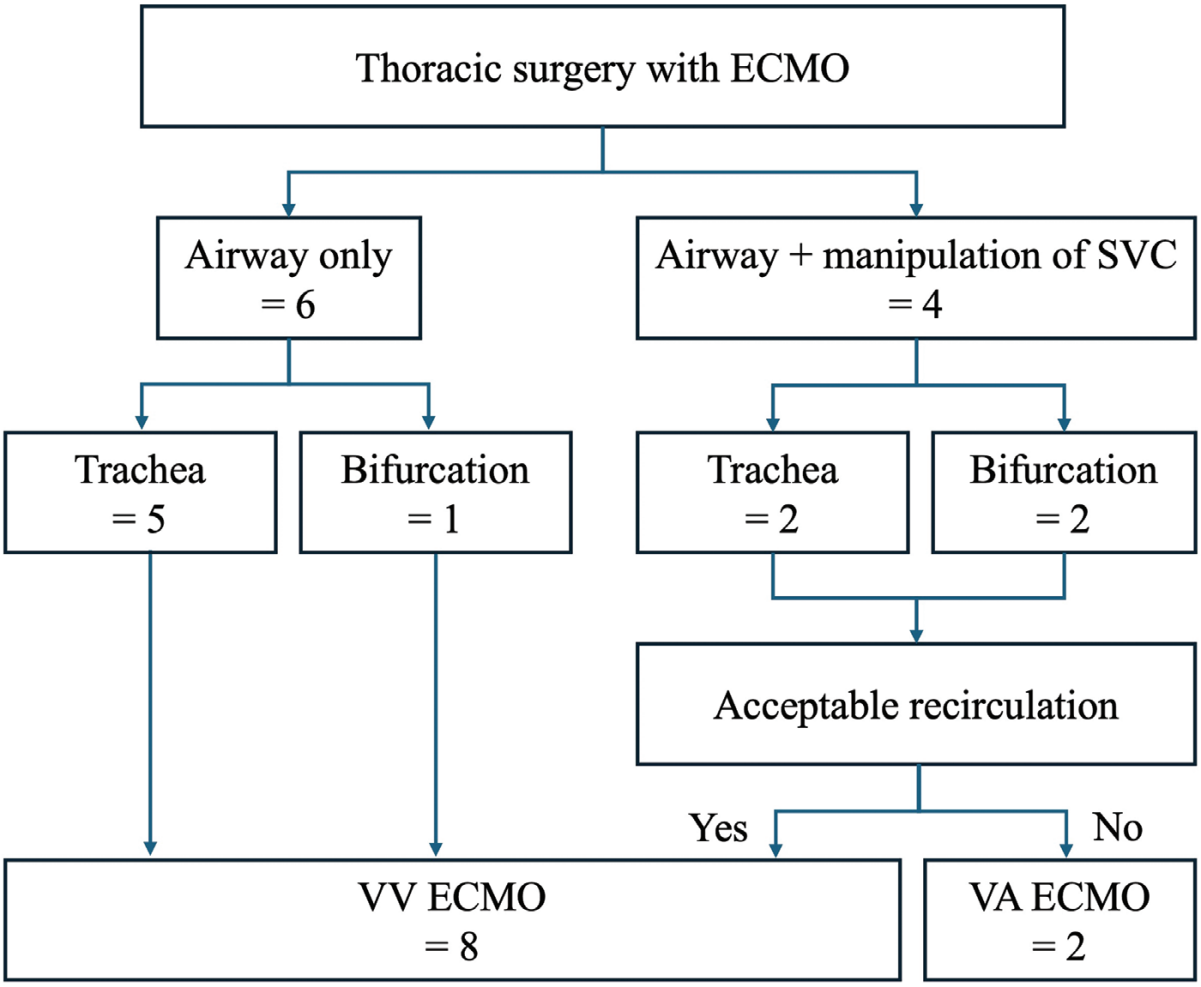
This figure outlines the criteria for ECMO selection for thoracic surgery at our institution. ECMO, extracorporeal membrane oxygenation; SVC, superior vena cava; VA, veno-arterial; VV, veno-venous

This case series documents 10 cases comprising 7 tracheal and 3 bifurcation surgeries (**Fig. 3**). We collected the following information: the patients’ baseline characteristics, surgery information, complications, matters related to ECMO, date and modality of recurrence, and survival period. VV-ECMO was used in 8 cases, while VA-ECMO was employed in 2 cases. **Table 1** lists the patients’ baseline characteristics, and **Table 2** lists the details of their surgeries and the settings of the ECMO. Six patients did not receive heparin maintenance and instead received nafamostat mesilate maintenance after a single dose of heparin or received only a single dose of heparin. Postoperative hemothorax occurred as one of the ECMO-related complications in Case 4. His complication was improved with noninvasive treatment alone. Tracheal/bronchial fistulas occurred in 3 cases (Case 1, 5, and 7) as the surgical complications. In Case 1, the patient needed to have over 5 cm trachea resected for radical surgery. Case 5 had poor general condition because of a G-CSF-producing lung cancer. He had anorexia, prolonged fever, and a high sustained systemic inflammatory response for 3 months before surgery. Case 7 underwent sleeve trachea-carinal resection and carinal reconstruction as emergency surgery. It remains unclear whether ECMO affected these surgical complications. There were no perioperative cardiopulmonary complications, in-hospital deaths, or deaths within 30 days after surgery. One patient died from metastatic recurrence of non-small cell lung cancer 5 months after surgery, another from progression of disease in mediastinal anaplastic cancer 4 months after surgery, and the 3rd from upper gastrointestinal bleeding 2 years after surgery. The other 7 patients remain alive.

**Table 1:** Patient characteristics, surgical procedures, and complications

No.	Age	M/F	Disease	Surgical procedure	Position	Admission status	ASA-PS	LVEF	Surgical complications	Survival period	Cause of death
1	57	M	Tracheal adenoid cystic carcinoma	Tracheal resection	Supine	Elective	1	64	Tracheoesophageal fistula	–	–
2	68	F	Granulation of metallic stent for tracheomalacia	Endobronchial argon plasma coagulation	Supine	Elective	1	84	–	–	–
3	69	M	Right upper lobe squamous cell carcinoma	Sleeve right upper lobectomy	Lateral	Elective	1	67.4	–	–	–
4	31	M	Tracheobronchial injury after blunt thoracic trauma	Tracheal bifurcation repair	Lateral	Bailout	1	70.3	–	–	–
5	62	M	Right upper lobe squamous cell carcinoma	Sleeve right upper middle lobectomy, resection, and reconstruction of superior vena cava	Lateral	Elective	1	72.5	Bronchopleural fistula	5 months	Metastasis of cancer
6	58	F	Inflammatory myofibroblastic tumor of the trachea	Tracheal resection	Supine	Elective	1	60.6	–	–	–
7	76	M	Tracheal bifurcation squamous cell carcinoma	Carinal resection	Lateral	Bailout	1	66.5	Aspiration pneumonia, intestinal pneumonia, and bronchopleural fistula	2 years	Upper gastrointestinal bleeding
8	78	F	Papillary thyroid cancer	Total thyroidectomy and tracheal resection	Supine	Elective	1	57.1	–	–	–
9	63	M	Mediastinal anaplastic cancer	Insertion of metallic stent	Supine	Bailout	1	70.1	–	4 months	Progression of cancer
10	66	F	Tracheal squamous cell carcinoma	Resection and reconstruction of trachea, internal jugular vein, esophagus, and bilateral recurrent laryngeal nerve	Supine	Elective	2	82.9	–	–	–

ASA-PS, American Society of Anesthesiologists-Physical Status; F, female; LVEF, left ventricular ejection fraction; M, male

**Table 2:** ECMO settings, anticoagulation, and complications

No.	ECMO	Cannulation				Blood flow		Body surface area (m^2^)	Anticoagulation		Heparin caring circuit	Operation time	ECMO introduction time	ECMO circulation time	Bleeding (mL)	ECMO complications
		Drainage	Fr	Return	Fr	L/min	mL/min/kg		Maintenance	Single dose**						
1	VV	LF*	25	RF	21	5.5	58.8	2.061	Heparin	Nafamostat	–	7:43	0:34	8:17	2795	–
2	VV	RI	21	RF	15	2.8	62.9	1.373	–	Heparin	–	0:16	1:00	1:16	58	–
3	VV	RF	25	LF	21	4.5	78.1	1.636	Nafamostat	Heparin	–	7:14	0:10	7:24	877	–
4	VV	LF	21	RI	21	5.9	55.8	1.742	Nafamostat	–	+	4:36	0:30	5:06	1181	Hemothorax
5	VA	RF	29	LF	18	4.5	80.4	1.648	Heparin	–	+	7:49	0:38	8:27	4101	–
6	VV	RF	25	RS	15	4.5	60.4	1.77	Heparin	–	–	3:43	0:17	4:00	15	–
7	VV	RF	25	RI	15	4	58.7	1.823	Heparin	–	–	8:28	0:25	8:53	430	–
8	VA	RF	21	RF	15	3	71.4	1.422	Nafamostat	Heparin	–	9:05	0:31	9:36	2189	–
9	VV	RF	24	RI	18	4.2	60.9	1.756	–	Heparin	+	1:36	0:21	1:57	8	–
10	VV	RF	21	RS	15	2.5	62.5	1.319	–	Heparin	+	12:05	0:25	12:30	3795	–

^**^Single dose: an initial administration of heparin at cannulation of ECMO.

ECMO, extracorporeal membrane oxygenation; F, femoral; I, internal jugular vein; L, left; R, right; S, subclavian; VA, veno-arterial; VV, veno-venous

### Case presentations

#### Case 1

**Fig. 4:**
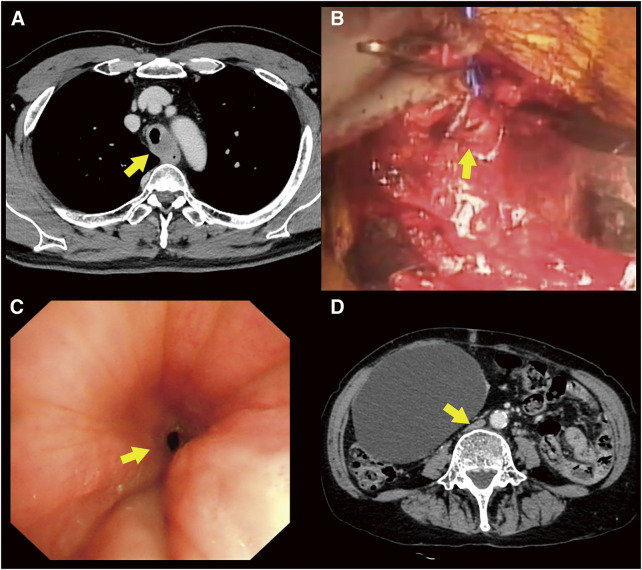
(**A**) Contrast-enhanced CT revealed a tumor 4.7 cm in length, extending cephalocaudally and dorsally to the trachea. A bronchoscopic biopsy confirmed an adenoid cystic carcinoma (arrow). (**B**) This intraoperative photograph of Case 1 shows that the dorsal half of the trachea has just been sutured (arrow) without the use of surgical field intubation. (**C**) Bronchoscopy in Case 2 revealed granulation stenosis (arrow) at the mouth side of the metallic stent inserted for tracheomalacia. (**D**) Contrast-enhanced CT in Case 2 showed compression of the inferior vena cava (arrow) by a large hepatic cyst. Because inserting a longer drainage cannula through the right femoral vein would be difficult, a drainage cannula was placed in the right internal jugular vein, and a return cannula was placed in the right femoral vein (VV-ECMO). CT, computed tomography; VV-ECMO, veno-venous-extracorporeal membrane oxygenation

A 57-year-old man presented with hoarseness. Contrast-enhanced CT revealed a tumor 4.7 cm in length, extending cephalocaudally and dorsally to the trachea. Bronchoscopic biopsy revealed an adenoid cystic carcinoma. Radiation therapy (40 Gy) was administered before surgery (**Fig. 4A**), followed by tracheal resection. It was decided that ECMO should be employed to reduce the frequency of intraoperative intubation. Because traction of the superior vena cava was anticipated, the drainage cannula was placed in the left femoral vein, and the return cannula was in the right femoral vein (VV-ECMO). Heparin administration was maintained during ECMO use. Surgery was performed through a median sternotomy with the patient in a supine position. The trachea was resected approximately 5 cm from the 2 rings on the mouth side of the bifurcation and reconstructed (**Fig. 4B**). The tumor had also invaded the esophagus, and a wedge resection was performed. The wound was closed with thymic fat and an intercostal muscle flap interposed between the tracheal anastomosis and the esophagus. After the surgery, the ECMO cannulas were removed, and protamine was administered. The patient has remained hospitalized until now for treatment of a tracheoesophageal fistula.

#### Case 2

The patient was a 68-year-old woman who presented with dyspnea about 7 months after tracheal stent insertion and a tracheostomy for tracheomalacia. Contrast-enhanced CT and bronchoscopy revealed granulation stenosis on the mouth side of the stent (**Fig. 4C**), and it was decided that argon plasma coagulation (APC) should be performed. It was anticipated that positive pressure ventilation would be difficult due to the tracheal stenosis. Therefore, the plan was to sedate the patient with dexmedetomidine and insert ECMO while the patient was awake. Because contrast-enhanced CT showed that the inferior vena cava was compressed by a large hepatic cyst (**Fig. 4D**), and it was thought that inserting a longer drainage cannula through the right femoral vein would be difficult, a drainage cannula was placed in the right internal jugular vein, and a return cannula was placed in the right femoral vein (VV-ECMO). After 3000 IU of heparin were loaded, the patient was managed heparin-free, and the highest intraoperative ACT value was 209 s. Surgery was performed with the patient in a supine position, and APC was performed using a flexible bronchoscope. After the surgery, the ECMO cannulas were removed, and the patient was discharged on the 7th postoperative day.

## DISCUSSION

In cases requiring thoracic tracheal and bifurcation surgery, ECMO can provide a tubeless surgical field for precise and rapid dissection as well as safe reconstruction. Conventional surgical field intubation, including cross-field ventilation, often results in poor visibility, and frequent interruptions in ventilation obstruct the surgical procedure.^18)^ Additionally, repeated and strong pressure applied to the resection stump may cause damage to the tracheal anastomosis and lead to ischemia.^19)^ Although high-speed jet ventilation can be used as an alternative to normal ventilation, its use has been associated with an increased risk of hypercapnia, barotrauma, and contralateral pneumothorax.^20)^ ECMO, on the other hand, prevents damage to the remaining lung and peripheral bronchus caused by operative field intubation. It also removes the time constraint on tracheal anastomosis by reducing the risk of unstable oxygenation. Additionally, peripheral structures can be directly assessed through visual inspection without interference from tracheal tubes. Distal tracheal anastomoses were also evaluated perioperatively using flexible bronchoscopy, which was safe and effective in ECMO-supported cases.

Moreover, ECMO is particularly beneficial in tracheal surgeries, enabling operations on patients who would otherwise be excluded due to their inability to intubate.

ECMO can be performed in 3 ways: venous drainage and venous return (VV), venous drainage and arterial return (VA), or venous drainage and both venous and arterial return (VVA). VV-ECMO assists oxygenation, VA-ECMO assists cardiac output even more, and VVA-ECMO is selected when oxygenation is poor with VA or VV-ECMO alone. However, a potential problem with VA-ECMO is that when returned blood collides with cardiac output, it can cause blood cell destruction and impede the delivery of oxygenated blood. In particular, poor oxygenation of cerebral blood and coronary arterial blood (north-south syndrome)^21)^ are complications that should be avoided. The likelihood of cerebral ischemia should be evaluated based on the anatomy of the circle of Willis, and intraoperative measurements of SpO_2_ in the right upper extremity and cerebral rSO_2_ should also be performed. In addition, 17% of patients experience leg ischemia related to femoral artery cannulation, with 5% requiring leg amputation.^22)^ Being under 20 years old and using a cannula larger than 20 Fr are risk factors.^23)^

ELSO guidelines^14)^ recommend an ACT-guided approach that aims for 1.5 times the normal ACT, achieved with undifferentiated heparin as the anticoagulant during ECMO use in non-bleeding patients. With that approach, approximately 40% of patients undergoing VV-ECMO experience bleeding or thrombotic events, and bleeding events are reportedly more associated with in-hospital mortality than thrombotic events (odds ratio 1.69 vs. 1.23).^10)^ Risk factors for bleeding events include acute kidney injury, vasopressor use before the start of ECMO, single-site cannulation, and prolonged ECMO. Risk factors for thrombotic events include cardiac arrest, heavy weight, high PaCO_2_ (>75 mmHg), and elevated pH (above the upper limit) before the start of ECMO, multi-site cannulation, and prolonged ECMO. As an alternative to heparin, nafamostat mesilate is often used in patients at high risk of bleeding or with heparin-induced thrombocytopenia. Nafamostat mesilate has a very short half-life (about 5–8 minutes) and is a broad-spectrum anticoagulant that can be used to selectively maintain high intra-circuit anticoagulant levels.^24)^ In addition, nafamostat mesilate may inhibit the inflammatory response triggered by contact with extracorporeal circuits, which suggests it may improve the prognosis of patients with ECLS. However, it should be noted that there is no antagonist for nafamostat mesilate, and it cause severe anaphylactic shock in rare cases.^25)^ Moreover, there have also been recent reports of heparin-free management after 3000 IU of heparin are loaded in patients with hemorrhage risk undergoing VV-ECMO.^11)^ This approach reduces bleeding events without increasing prognostic thrombotic events, and similar results have been reported for VA-ECMO.^26)^ These anticoagulation strategies should be tailored to the patient and the surgical risk.^27)^ In our cases, the anticoagulation strategy was determined by board-certified cardiovascular surgeons. Based on current evidence, a single dose of heparin is appropriate for intraoperative VV-ECMO if there is no risk of thrombosis.^11)^ However, in cases where there is a high risk of thrombosis and a low risk of bleeding, heparin or continuous administration of nafamostat mesilate (as an alternative anticoagulant to heparin) should be chosen. If the risks of both thrombosis and hemorrhage are high, the surgical indication itself should be carefully considered. Additionally, anticoagulation therapy, including heparin or nafamostat mesilate, will be essential when using long-time ECMO support after surgery.^28)^ Other concerns of thoracic surgeons include the risk of tumor distant metastasis and the induction of systemic inflammatory response syndrome (SIRS) from the use of the extracorporeal circuit. ECMO, being a closed circuit, does not use intraoperative blood salvaging autotransfusion, and blood around the tumor is not returned into the circuit. Therefore, it would have minimal effect on tumor metastasis.^29)^ Possible immune effects within 24 hours of extracorporeal circuit use include exposure of blood to surfaces in the circuit and activation of inflammatory cytokines and complement due to organ ischemia/reperfusion, leading to a systemic inflammatory response.^30,31)^ In VV-ECMO, the activation of complement and the coagulation system is relatively suppressed because cardiac output is maintained, blood circulation is pulsatile, and inflammatory components are removed as the blood flows through the lungs. Prolonged ECMO therapy can also lead to immunodeficiency if the above-mentioned reactions persist.^31)^ In this study, the patients did not experience any events, including SIRS or infections, directly related to ECMO. Perioperative ECMO can exacerbate postoperative SIRS; however, its frequency is uncertain, and further investigation will be needed.

## CONCLUSIONS

ECMO is useful for tracheal surgery and in cases of difficult or dangerous intubation because it makes safe and accurate surgery possible. Although the risk of catastrophic bleeding cannot be ignored in thoracic surgery, we believe that individualized anticoagulant strategies can be safely implemented. Careful advance planning with physicians from other departments and operating room staff is also important.

## ACKNOWLEDGMENTS

We thank Takashi Kumagai, a clinical engineer at Akita University Hospital, for his invaluable assistance in data collection.

## DECLARATIONS

### Funding

None.

### Authors’ contributions

All authors were involved in the surgery and management of these patients.

YH and KI were major contributors to the writing of the manuscript.

All authors read and approved the final manuscript.

### Availability of data and materials

Not applicable.

### Ethics approval and consent to participate

We obtained approval from the Institutional Ethics Committee of Akita University Hospital (Approval Number: 2679).

### Consent for publication

Written, informed consent was obtained from these patients for the publication of this case report.

### Competing interests

The authors declare that they have no competing interests.
